# Academic workforce trends in community hospitals

**DOI:** 10.3402/jchimp.v2i1.17361

**Published:** 2012-04-30

**Authors:** Britta L. Anderson, Jay Schulkin, Hal C. Lawrence

**Affiliations:** The American College of Obstetricians and Gynecologists, Washington, DC, USA

**Keywords:** Workforce, community hospital, ob-gyn, faculty, gender, university

## Abstract

**Introduction:**

Obstetrician-gynecologist faculty workforce studies have been limited to faculty at university training programs. Not much is known about the obstetrician-gynecologist faculty workforce at community programs.

**Method:**

This study assessed the obstetrician-gynecologist faculty workforce in community training programs via administering surveys to the department chairs. The questionnaire assessed number of current faculty by degree, work status (part-time/full-time), rank, and sub-specialty. Out of 125 programs, 65 responded (52% response rate).

**Results:**

The mean number of full-time faculty per department in community hospitals was 17 faculty. Two-thirds of community department chairs anticipated an increase in full-time faculty and 43% anticipated an increase in part-time faculty. Like university programs, sub-specialists and Professors (compared to generalists and assistant professors) were more likely to be male.

**Conclusion:**

There are similarities between the community and university faculty workforce, many of the community program faculty are involved in research. Given the evolving clinical, educational, and research demands on community faculty, it is important to continue to monitor and study community program faculty.

In 1975 a study of the academic departments of obstetrics-gynecology was conducted to assess the faculty workforce (1). Since that time, several follow-up studies have been performed to monitor the university academic workforce (2, 3). The most recent follow-up study was published in 2010 (4) and provided an insightful analysis of the longitudinal changes in the obstetrician-gynecologist workforce, mainly the increase in the number of women and part time faculty.

An important limitation of these series of studies is that community residency programs (programs that are not located in academic centers but may be affiliated with a university) were not included. Like university residency programs, community residency programs are training students and conducting research, however, these activities in community programs are often not acknowledged. Furthermore, community faculty are facing increasing demands on clinical productivity along with threats of graduate medical education budget cuts (5). There are roughly an equal number of community and university residency departments. This study was conducted to assess the workforce of residency training programs at community hospitals.

## Methods

### Sample

Community programs were identified from a list of residency programs. Surveys were administered to the chair of the departments or the residency program director.

### Survey

Respondents indicated the number of current faculty by degree, (MD/DO/PhD/other), work status (full-time/part-time/other paid/other unpaid), sub-specialty (MFM/gyn-oncology/reproductive endocrinology/ urogynecology/research only/other), and faculty rank (instructor/assistant professor/associate professor/professor). When providing these numbers, respondents indicated how many faculty in each category were male and female. This survey differentiated between full-time (1.0 full-time equivalent) and part-time (0.5–0.9 full-time equivalent) faculty. A definition of ‘faculty’ was not provided; each chair reported what they consider to be a faculty member. Respondents also provided demographic and department information.

### Data analysis

Data were analyzed using a personal computer based version of SPSS 17.0 (SPSS Inc., Chicago, IL, USA). Descriptive data were computed for primary analysis. *T*-tests were used to compare groups. Statistical significance was defined at ≤0.05 and at confidence intervals of 95%. Statistical comparisons were conducted between the most recent university sample data (4) and the data from this community samples.

## Results

### Community residency programs

A 52% response rate was obtained (65/125). Programs reported having an average of 4.7 (SD = 3.96) residents per year, with the range being 2 (*n* =5) to 24(*n* =1) (2 programs had 20 residents and the remaining had 8 or less). The mean number of total full-time faculty was 13.3 (SD = 11.0). On average, the mean number of MDs was 11.2 (SD = 8.5), the mean number of PhDs was 0.65 (SD = 2.5), and the mean number in the ‘other’ category was 0.8 (SD = 2.6). On average, departments had a ratio of 3.8 (SD = 2.4) MD faculty members (part-time or full-time) per one resident.

The number of paid faculty per department ranged from 1 to 61, with the average department consisting of 16.8 (SD = 11.3) faculty, median = 15, mode = 8. The range of unpaid faculty varied significantly with one department reporting as many as 60 unpaid female faculty (the other responses for female unpaid faculty were 1, 3, 10, and 15 and male unpaid faculty were 1, 2, 3, 5, 10, 16, 18, and 24).

Eighty three percent of departments (54 of 65) had part-time faculty. Of those departments, 34.0% of the faculty is part time on average. The number of part-time faculty ranged from 1 to 16.

Among departments with part-time females (*n* =53), the average percent of female faculty who are working part-time was 40% (SD = 35%); the median percent was 21%, the mode was 0% (15 programs had no part-time faculty). In 15% of departments, all of their female faculty are part-time.

Of the full-time faculty, 49.2% were women. Of the part-time faculty, 46.4% are women. Of the full-time ‘Other’ 80.8% were women and of the part-time ‘Other’, 61.1% were women. Among the ‘Other-paid’ physicians 44% were women, and ‘Other-unpaid’ physicians 56% were women.

Table 1 shows the mean and median number of faculty per department based on specialty type. A total of 56% of the responding programs have at least one specialist in all four of the following subspecialties: Maternal-Fetal Medicine, Gynecologic Oncology, Reproductive Endocrinology, and Urogynecology.

**Table 1:** Mean and median number of faculty per department based on specialty type

	Male		Female	
	
	Mean (SD)	Number of departments with that type of faculty	Mean (SD)	Number of departments with that type of faculty
General obstetrics-gynecology	5.76 (4.2)	55	6.67 (6.6)	52
Maternal-fetal medicine*	2.45 (1.3)	51	1.65 (1.1)	40
Gynecologic Oncology*	1.66 (0.84)	52	1.44 (0.5)	18
Reproductive Endocrinology*	1.76 (0.95)	46	1.50 (0.75)	28
Urogynecology*	1.45 (0.62)	33	1.10 (0.31)	20
Research only	1 (0)	5	1.16 (0.4)	6

* There were significantly more males than females in Maternal-fetal medicine (*t*=4.67, *p*<0.001), Gynecologic Oncology (*t*=6.2, *p*<0.001), Reproductive Endocrinology (*t*=3.8, *p*<0.001), and Urogynecology (*t*=3.3, *p*=0.002).

Table 2 shows the mean and median number of faculty per department based on rank. There were significantly more male Professors than female (*p*=0.02).

**Table 2:** Mean and median number of faculty per department based on instructor type among departments who have faculty.

	Male		Female	
	
	Mean (SD)	Number of departments with that type of faculty	Mean (SD)	Number of departments with that type of faculty
Instructor	4.62 (4.5)	24	5.82 (6.4)	28
Assistant Professor	4.40 (3.4)	45	4.26 (3.9)	42
Associate Professor	2.69 (3.2)	42	2.64 (2.7)	28
Professor*	2.31 (1.9)	35	1.18 (0.73)	17

* There were significantly more male than female Professors (*p*=0.02)

All but one program reported that they have faculty who are involved in research. About half (50.8%) indicated that 0–25% of their faculty are involved in research, 32.8% indicated 26–50% of faculty is involved in research, 14.8% indicated 51–75% of faculty is involved in research, and 1.6% indicated 76–100% of faculty is involved in research.

When asked how much time their full-time faculty spends doing research, 6.7% said no time, 33.3% said 5% of time, 48.3% said 10% of time, and 11.7% said 25% of time. When asked how much time their part-time faculty spends doing research, 37.3% said no time, 35.3% said 5% of time, 19.6% said 10% of time, and 7.8% said 25% of time.

### Comparison between university and community residency samples

On average, university residency programs had a greater number of total faculty (part-time and full-time MDs, PhDs, and Other paid faculty) than community residency programs (*p*<0.001). There was no significant difference in the mean number of part-time faculty, but, on average, university programs reported a greater number of full-time faculty (*p*<0.001). See Table 3.

**Table 3:** Mean and standard deviation of the number of paid faculty at university and community residency programs. University data obtained from Rayburn et al. (4)

	University	Community
Total*	35.8 (SD = 28.7)	16.8 (SD = 11.3)
Full-time*	29.9 (SD = 23.2)	12.6 (SD = 10.8)
Part-time	6.6 (SD = 11.8)	4.2 (SD = 4.1)

* *p*<0.001

University residency programs had a greater mean number of full-time MDs and PhDs than community residency programs (all *p*<0.001), but there was no difference in part-time MDs or PhDs. While there was not a significant difference in the mean number of male faculty who practiced general ob-gyn, there was significantly more female faculty who practiced general ob-gyn in university programs than community programs (*p*=0.013). There was significantly more faculty in the university programs for all sub-specialties except male uro-gynecologists. There was no mean difference in the number of male or female instructors between the university programs and the community programs.

## Discussion

When comparing the results with university programs (4), it was found that community programs are generally smaller than university programs. Previous research suggests that there are few differences in the inpatient training (e.g., number of diagnoses, types of diagnoses, etc) between community programs and university programs (6). Similarly, a comparison of demographics, prior training, and past experiences of the faculty found very few differences in community and university training programs (7).

Though university faculty were found to spend more time in research than community faculty (7), the results of this study support that conducting research is common in community residency programs. All but one program reported that they have faculty who are involved in research and over half of the programs indicated that more than 25% of their faculty are involved in research.

The trends among male and female obstetricians and gynecologists were similar among the university and community studies. The disproportionate number of males in higher ranked positions in both university and community programs is likely to change as females now comprise 70% of those medical residents specializing in obstetrics and gynecology, which was previously a male dominated specialty.

While roughly the same percentage of university (84%) and community (83%) programs had part-time faculty, community programs reported that a larger percentage of their faculty are currently part-time. This difference may be due to the fact that when community programs were established with primarily part-time unpaid volunteers from private practice (8).

In conclusion, this is only the first look at community residency program faculty workforce. Half of residency programs are community based and these programs train approximately 40% of all ob-gyn residents. With increasing pressures for clinical productivity (9) and increasing demands for trainee supervision (5), medical faculty are finding it increasingly more difficult to balance research, education, and clinical practice (the three principles of academic medicine that Flexner called the ‘three-legged stool’). As academic medicine continues to evolve under these changing pressures, further research should continue to monitor the community faculty workforce in order to detect changes that might impact the quality of medical training.
